# Plasma N-terminal tau fragment levels predict future cognitive decline and neurodegeneration in healthy elderly individuals

**DOI:** 10.1038/s41467-020-19543-w

**Published:** 2020-11-27

**Authors:** Jasmeer P. Chhatwal, Aaron P. Schultz, Yifan Dang, Beth Ostaszewski, Lei Liu, Hyun-Sik Yang, Keith A. Johnson, Reisa A. Sperling, Dennis J. Selkoe

**Affiliations:** 1 Massachusetts General Hospital, Boston, MA USA; 2 Brigham and Women’s Hospital, Boston, MA USA; 3 Harvard Medical School, Boston, MA USA

**Keywords:** Cognitive ageing, Neurological disorders

## Abstract

The availability of blood-based assays detecting Alzheimer’s disease (AD) pathology should greatly accelerate AD therapeutic development and improve clinical care. This is especially true for markers that capture the risk of decline in pre-symptomatic stages of AD, as this would allow one to focus interventions on participants maximally at risk and at a stage prior to widespread synapse loss and neurodegeneration. Here we quantify plasma concentrations of an N-terminal fragment of tau (NT1) in a large, well-characterized cohort of clinically normal elderly who were followed longitudinally. Plasma NT1 levels at study entry (when all participants were unimpaired) were highly predictive of future cognitive decline, pathological tau accumulation, neurodegeneration, and transition to a diagnosis of MCI/AD. These predictive effects were particularly strong in participants with even modestly elevated brain β-amyloid burden at study entry, suggesting plasma NT1 levels capture very early cognitive, pathologic and neurodegenerative changes along the AD trajectory.

## Abstract (web summary)

Previously it was shown that an N-terminal tau fragment (NT1) measured in plasma can differentiate individuals with Alzheimer’s disease from healthy controls. Here the authors show that plasma levels of NT1 can associate with future cognitive decline in cognitively normal elderly individuals.

## Introduction

Biofluid markers of neurodegeneration, proteinopathy, and neural injury have great potential to elucidate mechanisms underlying neurodegenerative disease progression and accelerate the development of experimental therapeutics. Biofluid measures that detect pathologic changes which precede clinical symptoms may be particularly useful, as targeting preclinical stages of disease will enable intervention prior to widespread neural and synaptic loss. Preclinical stages of Alzheimer’s disease (AD) are characterized by elevated brain β-amyloid (Aβ) burden as assessed by PET imaging, cerebrospinal fluid (CSF) testing, and most recently, by biochemical analyses of blood^1–5^. As a group, those with evidence of elevated brain β-amyloid burden generally show greater rates of cognitive decline, but numerous cohort studies also demonstrate that many individuals with elevated brain β-amyloid do not show clear cognitive decline, at least during the available follow-up^6–8^. This highlights the particular need to develop non-invasive disease biomarkers that are predictive of impending cognitive decline and neurodegeneration, alone or in concert with measures of β-amyloid. This goal is especially important in the context of AD clinical trials, where the move toward early intervention presents a problem: reliably identifying participants at high risk of neurodegeneration and cognitive decline over the course of a relatively short clinical trial.

Advances in the detection of biofluid measures of AD pathology and neurodegeneration, including blood-based and CSF measures of total tau^9^, phosphorylated tau species (including phosphorylation sites at position 181 and 217)^5,10–13^, and neurofilament light chain (NfL)^14–19^ have the potential to bridge this gap and provide complementary information to that provided by biomarkers of β-amyloid burden. In addition to broadening our understanding of tau pathologic burden and characterizing in-progress neurodegeneration, the availability of inexpensive, blood-based tau biomarkers would allow for much larger-scale screening than is currently possible using invasive techniques or expensive imaging modalities.

Recent work demonstrates that a newly identified N-terminal tau fragment (NT1) measured in plasma can differentiate normal, mildly-impaired, and AD dementia populations with high specificity and sensitivity in discovery and replication cohorts^20^. This prior work demonstrated that the NT1 fragment measured in CSF or blood discriminated AD and non-AD populations substantially better than full-length tau and tau measured via commonly used, mid-region directed antibodies alone. Coupled with findings demonstrating that N-terminal containing tau fragments may be actively secreted from neurons when exposed to Aβ (as opposed to passively released in response to general cell injury, as occurs with some other tau species)^21,22^, the work to date suggests that N-terminal containing tau fragments may provide particular insight into early Aβ-driven neurodegenerative processes^20,22^. In this context, we examine the extent to which alterations in plasma NT1 can actually predict future cognitive decline and neuronal injury and loss along an AD trajectory in cognitively unimpaired older adults. To do this, we systematically examined whether plasma NT1 predicts prospective cognitive change, neurodegeneration, and tau accumulation in a well-characterized cohort of cognitively normal older adults participating in the Harvard Aging Brain Study (HABS). We went on to compare NT1 and NfL as predictors of cognitive and neurodegenerative trajectories and of AD pathologic changes.

## Results

### Baseline plasma NT1 levels predict future cognitive decline in clinically normal elderly

NT1 and NfL were measured in blood plasma samples obtained during the first year of HABS using ultrasensitive, single-molecule array technology (Quanterix Simoa; see “Methods”)^15,23,24^. Plasma measures were compared with cognitive performance (Preclinical Alzheimer’s Cognitive Composite^25,26^; PACC5), with PET measures of β-amyloid (11C-Pittsburgh Compound B; PiB) and tau (18F-Flortaucipir; FTP), and with hippocampal and total gray matter volume, all derived using previously validated protocols^27–29^ (“Methods”; Table 1).

**Table 1:** Study sample demographics.

	Main sample	Tau dataset
No. of participants (M/F/total)	94/142/236	62/50/112
Mean age (±SD)	73.57 ± 6.09 yr	72.73 ± 6.03 yr
Median follow-up (±SD)	5.04 ± 0.99 yr	2.41 ± 1.31
Mean # of MRIs (range)	2.22 (1–4)	NA
Mean # of FTP PET (range)	NA	2.32 (2–4)
APOE **ε**4 status (**ε**4−/**ε**4+)	169/67	80/32
Median years of education	16 (8–20)	17 (8–20)

**Fig. 1:**
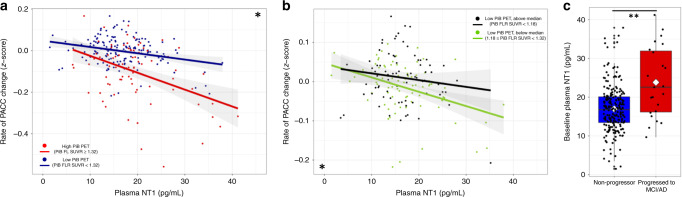
Higher baseline NT1 is associated with greater cognitive decline and progression to clinical impairment, alone and synergistically with greater β-amyloid burden. Greater levels of plasma NT1 were associated with greater performance declines on the preclinical Alzheimer’s cognitive composite (PACC5; **a** and **b**, both *n* = 236). Using previously defined cutoffs for high (PiB FLR SUVR ≥ 1.32; **a**: red line and circles) and low (PiB FLR SUVR < 1.32; **a**: blue line and circles) PiB groups, we observed that greater plasma NT1 at study baseline was associated with cognitive decline in both groups, though this association was stronger in the high PiB group (**a**; high PiB: *t*(74) = −3.09, *p* = 0.008; low PiB: *t*(151) = −3.00, *p* = 0.008). Focusing on only the low PiB group, plasma NT1 levels interacted with higher levels of PiB (**b**: green; PiB FLR SUVR between 1.18 and 1.32; *t*(75) = −3.5, *p* = 0.0025) to predict decline, but this same effect was not seen in participants with PiB FLR SUVR values below the median of the low PiB group (**b**: black; PiB FLR SUVR < 1.18; *t*(70) = 0.06, *p* = 0.95). Within this longitudinally-followed sample (*n* = 236), 23 participants progressed to clinical impairment during the follow-up period. Individuals who progressed to MCI or AD dementia during follow-up had greater plasma NT1 levels than those who did not progress to clinical impairment (**c**: blue: non-progressors; red: progressed to MCI or AD; white diamond corresponds to group mean; *t*(230) = −4.03, *p* = 0.0003). Shaded regions represent 95% confidence intervals for the fit of each linear regression model. * and ** corresponds to FDR corrected *p* ≤ 0.005 or 0.0005, respectively, for the association of baseline NT1 to longitudinal PACC performance (**a**, **b**) or to progression to clinical impairment during follow-up (**c**), after correction for age, sex, APOE ε4 status, and years of education. Two-tailed *t*-tests were used throughout. For the boxplot in panel **c**, the bounds of the 25th and 75th percentiles are represented in the box, whiskers correspond to +/−1.5 times the interquartile range, line indicates the median, diamond indicates the group mean, and all data points are shown.

We observed that baseline NT1 levels were significantly associated with baseline cognitive function as measured by the PACC5, in that greater baseline plasma NT1 correlated with poorer cognitive performance at study entry (*t*(230) = −2.65, *p* = 0.015). Greater baseline NT1 levels were also strongly associated with more steeply negative PACC5 slopes during longitudinal follow-up (median 5.04 +/− 0.99 yr) in linear models (*t*(230) = −5.37, *p* = 0.003; Cohen’s *d* = −0.71, Fig. 1, Supplementary Table 1), as well as with poorer longitudinal PACC5 performance in linear mixed-effects models (*t*(1070) = −5.05, *p* < 0.0005).

Correcting for demographic covariates, the magnitude of the association of baseline NT1 (Cohen’s *d* = −0.71) with the slope of cognitive decline was greater than that seen for baseline hippocampal volume (HV; *d* = 0.61; Supplementary Tables 1 and 2) and only slightly weaker than baseline PiB PET (*d* = −0.91; Supplementary Table S1-2). Notably, the association of baseline plasma NT1 with cognitive decline remained similarly robust even after simultaneously correcting for PiB PET and hippocampal volume (*d* = −0.63, *p* < 0.0005 with correction for these measures; Supplementary Table 2), indicating that NT1 is capturing variance relevant to cognitive decline that is largely independent of these well-established AD biomarkers.

Though a relatively small number of participants in this initially cognitively normal sample progressed to clinical states of impairment (23 of 236, 9.7%; 20 to Mild Cognitive Impairment and 3 to AD dementia), we observed that higher plasma NT1 levels at study baseline were strongly associated with clinical progression (*t*(230) = −4.03, *p* < 0.0005; corrected for age, sex, ApoE ε4 status, and length of follow-up; Fig. 1c). We further observed that upon dividing all study subjects into tertiles based on NT1 levels, an increase in one tertile in baseline plasma NT1 by itself was associated with a 2.4-fold increased risk (OR 2.40; 95%CI: 1.35–4.63 95%CI) of progression to MCI or AD dementia during longitudinal follow-up. Consistent with results using the PACC5 cognitive composite, NT1 remained a significant predictor of progression to MCI or AD dementia even after correcting for demographic covariates, baseline PiB PET, and HV (*t*(228) = 2.93, *p* = 0.008).

### NT1 levels are synergistic with β-amyloid burden in predicting future cognitive decline

To examine potential interactions between β-amyloid burden and NT1 with respect to cognitive decline, we performed these same models stratifying participants into high and low PiB groups based on baseline amyloid PET, using cutoff values previously defined in the HABS sample^6,29^. We observed that baseline NT1 level was predictive of the rate of PACC5 change in both high and low PiB groups (high PiB: *t*(74) = −3.09, *p* = 0.008, Cohen’s *d* = 0.72; low PiB: *t*(151) = −3.00, *p* = 0.008, Cohen’s *d* = 0.49; Fig. 1a). We observed a significant interaction of PiB group and baseline NT1 with respect to PACC5 slope, indicating that the association of NT1 with PACC5 slope was more pronounced in high PiB versus low PiB participants (PiB group by NT1 interaction: *t*(228) = −2.71, *p* = 0.013). A similar PiB by NT1 interaction was seen using PiB as a continuous rather than dichotomous measure (PiB by NT1 interaction: *t*(228) = −3.65, *p* = 0.0012).

We performed two additional analyses to examine the association of NT1 with cognitive decline in the low PiB group. First, we performed a median split of PiB FLR SUVR values in only the low PiB group and observed that baseline plasma NT1 was predictive of cognitive decline in those above the median for the low PiB group (i.e., PiB FLR SUVR values between 1.18 and 1.32; *t*(75) = −3.5, *p* = 0.0025) but not in those below the median for the low PiB group (i.e., PiB FLR SUVR values between 1.00 and 1.179; *t*(70) = 0.06, *p* = 0.95). Second, we performed a floodlight (Johnson–Neyman) analysis^30–32^ to determine the threshold PiB FLR SUVR at which the NT1 by PiB interaction became a significant predictor of cognitive decline. Using this approach, the threshold at which the NT1 begins to interact with PiB to predict cognitive decline is a PiB FLR SUVR value of 1.16, well below the threshold to be included in the high PiB group using our conventional threshold for amyloid positivity (PiB FLR SUVR = 1.32; Fig. 1b, Supplementary Fig. 1). Both of these analyses suggest that the pathobiological processes represented by elevated plasma NT1 levels act synergistically with even relatively low, sub-threshold levels of β-amyloid to promote cognitive decline (Fig. 1; Supplementary Fig. 1).

### NT1 predicts decline across multiple cognitive domains, especially episodic memory

**Fig. 2:**
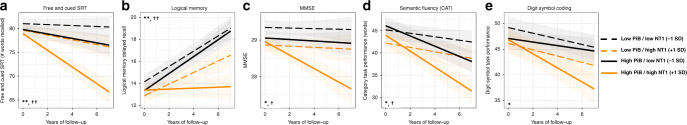
Baseline NT1 predicts longitudinal change on all components of the PACC. NT1 associations with cognitive performance were seen across all of the measures that comprise the PACC5. Individuals with both high NT1 (1 SD above the group mean; orange lines) and high PiB (mean value for the high Aβ group; solid lines) showed the greatest decreases in cognitive performance—especially in measures of episodic memory (**a**: *t*(1044) = −4.47, *p* = 0.00017; **b**: *t*(1070) = −3.92, *p* = 0.0005), but also on the MMSE (**c**; *t*(1070) = −2.69, *p* = 0.0013) and measures of language (**d**; *t*(1056) = −2.08, *p* = 0.05) and executive function (**e**; t(1069) = −2.05, *p* = 0.05). **, * corresponds to FDR corrected *p* ≤ 0.001 and ≤0.05, respectively, for the association of baseline NT1 to each longitudinal cognitive measure after correction for age, sex, APOE ε4 status, years of education; ††, † corresponds to FDR corrected *p* ≤ 0.01 and ≤0.05, respectively, for the interaction of baseline NT1 and PiB PET to each longitudinal cognitive measure after correction for age, sex, APOE ε4 status, and years of education. Two-tailed *t*-tests were used throughout. Shaded regions represent 95% confidence intervals for predictions based on each linear regression model.

To examine whether decline in particular cognitive domains may be differentially associated with baseline NT1 levels, we decomposed the PACC5 into its constituent measures and examined longitudinal associations with NT1 in exploratory, post hoc analyses. These analyses indicate that higher plasma NT1 levels are significantly associated with greater decline in all cognitive tests comprising the PACC5—especially with tests of episodic memory known to be highly sensitive to early AD-related cognitive decline (Free and Cued Selective Reminding Task: *t*(1044) = −4.47, *p* = 0.00017; Logical Memory Delayed Recall: *t*(1070) = −3.92, *p* = 0.0005; Fig. 2a, b), but also with a widely used cognitive composite (Mini-Mental State Status Exam: *t*(1070) = −2.69, *p* = 0.0013; Fig. 2c), a test of language function (Category Semantic Fluency: *t*(1056) = −2.08, *p* = 0.05; Fig. 2d), and a test of executive function and speed-of-processing (Digit Symbol Coding Task: *t*(1069) = −2.05, *p* = 0.05; Fig. 2e). Notably, as a strong practice effect is present in Logical Memory Delayed Recall performance, a diminished practice effect rather than a decline in raw performance was seen in association with high NT1 and PiB levels. Though follow-up studies with more focused testing within each cognitive domain are needed, the association of higher NT1 levels with poorer performance across all components of the PACC suggests that plasma NT1 may be predictive of worsened longitudinal cognition across a broad set of cognitive domains, especially memory.

### NT1 levels interact with β-amyloid burden to predict future neurodegeneration

**Fig. 3:**
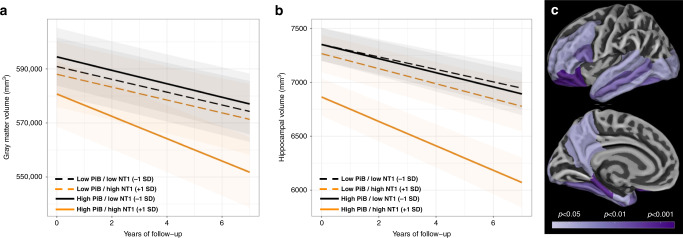
Higher baseline NT1 is associated with greater longitudinal neurodegeneration as measured by longitudinal structural MRI, particularly in those with high β-amyloid at baseline. Greater levels of plasma NT1 were associated with greater longitudinal declines in total gray matter volume (**a** *t*(291) = −2.16, *p* = 0.039) and hippocampal volume (**b** *t*(292) = −2.71, *p* = 0.013). These associations were present in individuals with both elevated (solid lines) and non-elevated levels of β-amyloid (dashed lines) assessed by PiB PET at study baseline, but the relationship of higher NT1 to neurodegeneration measures was particularly strong in individuals with higher amyloid burden (orange solid line). High (orange lines) and low NT1 (black lines) correspond to 1 SD above and below the group mean. High (solid lines) and low PiB (dashed lines) corresponds to the mean value for high PiB and low PiB individuals based on previously published cutoff values for PiB PET. An exploratory analysis of regions where the interaction of baseline NT1 and baseline PiB PET was significantly associated with regional cortical thickness is shown in panel (**c**), with nominal *p*-values indicated on the color scale. * corresponds to FDR corrected *p* < 0.05 for the association of NT1 with neurodegeneration; † to FDR corrected *p* < 0.05 for the interaction of NT1 and PiB group with respect to neurodegeneration. Two-tailed *t*-tests were used throughout. Shaded regions represent 95% confidence intervals for predictions based on each linear regression model.

Following examination of these NT1 associations with cognition, we assessed relationships between NT1 and MRI-based measures of neurodegeneration, including total gray matter volume (GMV) and hippocampal volume (HV). Greater baseline NT1 was not associated with lower baseline GMV (*t*(230) = −1.15; *p* = 0.28) but was associated with greater longitudinal decreases in GMV during follow-up imaging (*t*(291) = −2.16, *p* = 0.039; Fig. 3a). Similarly, baseline NT1 was not significantly associated with baseline HV (*t*(230) = −1.30, *p* = 0.23), but greater baseline NT1 was significantly associated with greater HV loss during longitudinal follow-up (*t*(292) = −2.71, *p* = 0.013; Fig. 3b). A significant interaction of baseline β-amyloid burden (PiB PET) with baseline NT1 as regards tissue volume was observed, such that individuals with elevated PiB PET and high NT1 at entry showed greater declines in both GMV (NT1 by PiB interaction: *t*(290) = −2.64, *p* = 0.016) and HV (NT1 by PiB interaction: *t*(290) = −2.40, *p* = 0.028; Fig. 3).

Exploratory, post hoc analyses examined associations between regional cortical thickness changes and baseline NT1 levels to elucidate the anatomic pattern of neurodegeneration associated with higher plasma NT1. In individuals with high PiB burden at study entry, greater baseline plasma NT1 was significantly associated with greater cortical thinning in several medial and lateral temporal regions of interest, as well as in frontal regions (Fig. 3c). In contrast, greater baseline NT1 was not significantly associated with greater cortical thinning in individuals with below-threshold (i.e., PiB PET SUVR < 1.32 at study entry) levels of PiB (all regions examined *p* > 0.05).

### NT1 levels are associated with tau accumulation in individuals with elevated β-amyloid

**Fig. 4:**
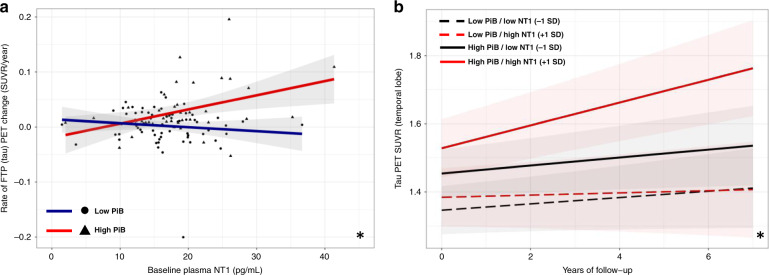
Greater plasma NT1 is associated with greater increase in temporal tau PET signal in individuals with high β-amyloid burden. In a subset of individuals with plasma NT1 and longitudinal FTP tau PET, greater levels of plasma NT1 at baseline were significantly associated with greater longitudinal increases in tau PET signal in the temporal composite region of interest. Panel **a** (*n* = 112) depicts the relationship of plasma NT1 to longitudinal FTP change in the temporal composite (NT1 by PiB interaction, *t*(141) = 2.78, *p* = 0.012). Panel (**b**) depicts the modeled change in temporal tau PET signal in individuals with high (red solid line; mean of high PiB group) and low PiB PET (black solid line; mean of low PiB group) and high (red lines; 1 SD above group mean for NT1) and low NT1 (black lines; 1 SD below group mean for NT1). Two-tailed *t*-tests were used throughout. Shaded regions represent 95% confidence intervals for the fit of each linear regression model in (**a**) and for model predictions in (**b**) * corresponds to FDR corrected *p* < 0.01 for the interaction of PiB and NT1 after correction for age, sex, and APOE ε4 status.

Data from participants with available NT1, PiB (amyloid) PET, and longitudinal FTP (tau) PET were used to assess possible relationships between NT1 levels and tau neurofibrillary pathology (Table 1; Tau PET dataset). As expected, higher baseline PiB PET alone predicted greater FTP PET signal increase over time (*t*(107) = 3.60, *p* = 0.013). In this subset, plasma NT1 levels were not cross-sectionally associated with a cortical composite of PiB PET signal or with a temporal composite of FTP PET signal after correction for age, sex, and APOE ε4 status (PiB: *t*(107) = 1.00, *p* = 0.34; FTP: *t*(107) = 0.911, *p* = 0.38). However, higher NT1 levels at the time of initial FTP PET were associated with greater longitudinal increases in temporal FTP PET signal in participants with elevated β-amyloid burden (NT1 by PiB interaction, *t*(141) = 2.78, *p* = 0.012; effect of NT1 in the high PiB group: *t*(55) = 2.27, *p* = 0.039; Fig. 4). Post hoc exploratory regional analyses of these same data suggest that several temporal and parietal FreeSurfer-defined cortical regions show a similar interaction of plasma NT1 and PiB PET with respect to longitudinal FTP PET signal (Supplemental Fig. 3).

### Plasma NT1 level is a stronger predictor of cognitive decline and neurodegeneration than NfL

**Fig. 5:**
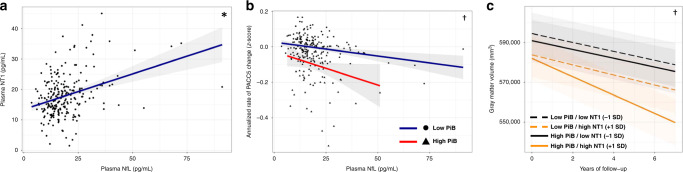
Plasma NfL levels are correlated with plasma NT1, longitudinal cognitive decline, and longitudinal GM volume. NT1 and NfL in the same plasma samples were significantly associated with each other (**a**; *n* = 236; *t*(230) = 4.39, *p* = 0.0030). Greater baseline plasma NfL was associated with greater cognitive decline in individuals with high PiB PET at baseline (**b**; *n* = 236; *t*(1074) = −2.14, *p* = 0.046). Greater baseline plasma NfL was associated with greater gray matter volume loss in individuals with higher baseline β-amyloid as assessed by PiB PET (**c**; *t*(290) = −2.768, *p* = 0.0129). * corresponds to FDR corrected *p* < 0.05 for the main effect of NT1 after correction for age, sex, and APOE ε4 status; † corresponds to FDR corrected *p* < 0.05 for the interaction of NfL and PiB after correction for age, sex, APOE ε4 status, and years of education. Two-tailed *t*-tests were used throughout. Shaded regions represent 95% confidence intervals for the fit of each linear regression model in (**a**) and (**b**) and for model predictions in (**c**).

NfL levels in cerebrospinal fluid (CSF) and plasma have come into increasing use as biofluid markers potentially reflective of ongoing neurodegeneration and/or neuronal injury^23,33–35^. We next assessed NfL in the same baseline plasma samples to provide context for the observations with NT1 described above. Comparing biomarker values quantified in the baseline plasma samples, we observed a significant correlation between NT1 and NfL levels after correction for age, sex, and APOE ε4 status (*t*(230) = 4.39, *p* = 0.0030; Fig. 5a). Both baseline NT1 and NfL were significantly correlated with age, but this association was stronger for NfL as compared to NT1 (NfL: *r* = 0.439, *t*(234) = 7.47, *p* < 0.0005; NT1: *r* = 0.239, *t*(234) = 3.77, *p* = 0.0009; Supplemental Fig. 2). Baseline NfL was not related to baseline PiB, HV, or gray matter volume (all *p* > 0.4). In the subset of participants with available tau PET within 1 year of NfL assessment, no relationship was seen between NfL and tau PET signal in the temporal composite (*t*(107) = 1.34, *p* = 0.222).

With respect to cognition, baseline plasma NfL levels were not correlated with baseline PACC5 performance (*t*(230) = 0.066, *p* = 0.95), in contrast to plasma NT1. Similar to NT1, baseline NfL interacted significantly with baseline PiB to predict PACC5 decline (*t*(1074) = −2.14, *p* = 0.046; Fig. 5b). However, when both a NfL by PiB interaction and a NT1 by PiB interaction were included in the same model predicting cognitive change, only the NT1 interaction with PiB remained significant (NT1*PiB, *t*(1067) = −4.36, *p* < 0.0005; NfL*PiB *t*(1067) = 1.19, *p* = 0.268). Similar to NT1, NfL at study baseline interacted with higher baseline PiB PET to predict greater GMV loss during longitudinal follow-up (*t*(290) = −2.768, *p* = 0.0129; Fig. 5c). A trend-level relationship between baseline NfL and longitudinal HV was observed after correction for age, sex, and ApoE ε4 status (*t*(292) = −1.73, *p* = 0.106).

## Discussion

Here, we investigated a N-terminal fragment of the tau protein (NT1) measured in plasma as a potential predictor of future cognitive decline, neurodegeneration, tau accumulation, and the development of MCI or AD in clinically normal elderly. We observed that greater plasma NT1 measured at entry into a longitudinal study was associated with significantly greater prospective decline in cognitive performance, especially in measures of episodic memory, but also in measures of executive function and semantic memory. Similarly, baseline plasma NT1 levels were significantly higher in participants who progressed to a stage of clinical impairment during longitudinal follow-up. MRI-based measures of neurodegeneration indicated that greater baseline plasma NT1 was associated with steeper declines in total gray matter and hippocampal volume during follow-up. We also observed that higher plasma NT1 predicted greater increases in temporal cortex tau burden during longitudinal follow-up in individuals with preclinical AD. In this cohort of older individuals who were clinically normal at baseline, plasma NT1 appeared to be a stronger predictor of cognitive and neurodegenerative trajectories than a widely used biofluid marker of neurodegeneration, NfL.

Though baseline plasma NT1 was not related to baseline PET measures of β-amyloid burden, the interaction of baseline plasma NT1 and baseline PiB PET was observed to be a particularly strong predictor of subsequent tau accumulation, as well as cognitive and neurodegenerative changes. The observation that plasma NT1 is predictive of longitudinal tau accumulation but is less well associated with cross-sectional tau raises the intriguing possibility that plasma NT1 may capture nascent tau pathology that is not yet easily detected by tau PET. This would be similar to what has been observed with CSF measures of Aβ, where alterations in Aβ levels can precede elevated β-amyloid burden visible on PET^36–38^. This would also be consistent with the expectation that tau pathology in a clinically normal population such as HABS would be at a very early stage, at a point where tau PET may not yet be abnormal. Further longitudinal follow-up with tau PET and studies in symptomatic populations are needed to test this hypothesis and clarify whether elevations in plasma NT1 may occur prior to observable changes on tau PET.

Technological advances in the sensitivity and reproducibility of biochemical analyses of biofluid analytes have yielded potential biomarkers of AD-related pathology, neurodegeneration, and neuroinflammation. Biomarkers sensitive to the earliest AD-related cognitive and neurodegenerative changes have the potential to improve risk stratification in a critical clinical trial population—older adults who are in the preclinical phase of the AD trajectory, prior to clinically evident cognitive impairment, synaptic compromise, and catastrophic neurodegeneration. This is particularly true of blood-based biomarkers, which are relatively non-invasive, cost-effective, and far more practical to measure longitudinally than serial PET imaging or CSF studies. Given recent, promising findings seen with emerging plasma assays for tau and phospho-tau species^9–11,39^, efforts to standardize and automate these assays across laboratories are the next critical step toward realizing the broad clinical and research utility of these measures. The NT1 assay employed here makes use of widely available antibodies, is now in use in multiple research groups, and has been successfully transitioned to highly sample volume-efficient and reproducible platforms (Quanterix Simoa HD-1 (bead-based) and recently to the plate-based Quanterix SP-X). These features of the NT1 assay make it well-suited for standardization, automation, and replication across different laboratories and cohorts.

We observed that correlations between NT1 and longitudinal PET measures of tau pathology were present in high PiB individuals, suggesting that high NT1 levels may predict the progression of tau pathology in those on an AD trajectory. Additionally, though NT1 levels were predictive of longitudinal cognitive (PACC) and neurodegenerative trajectories (HV, GMV) in both high and low PiB groups, NT1 effects were stronger when interacted with PiB binding or restricting the analysis to the high PiB group (Figs. 1 and 2). Critically, secondary analyses of the effects of NT1 in the “amyloid-negative” (Low PiB PET) group suggest that the pathobiological processes reflected in elevated plasma NT1 levels interact with even relatively low levels of β-amyloid to predict the risk of cognitive decline. This pattern of interactions between NT1 and even low β-amyloid burden suggests that plasma NT1 may be particularly useful in clinical research settings where identifying individuals in the earliest stages of the AD trajectory is crucial. These results are also consistent with prior studies demonstrating interactions between CSF measures of tau and Aβ42 for diagnostic purposes^40,41^.

The results here add to the rapidly emerging literature on plasma tau measures in AD clinical research. Nearly all of the elegant recent work on tau biomarkers has focused on phospho-tau species, particularly tau T181P and T217P^5,10–13^. Together with prior reports^20,22^, the findings here highlight that, in addition to the phosphorylation state of plasma tau, the differential truncation patterns observed in circulating tau fragments may themselves capture important aspects of AD-related neurodegeneration and cognitive decline (even very early in the course of the disease). Recent work demonstrates that the bulk of extracellular tau species are not full-length tau, but rather a variety of C- and N-terminally truncated forms^20,22^, a diversity that was not fully recognized previously, as the most commonly used tau assays targeted only mid-peptide tau epitopes. These different tau fragments likely have differential relationships to common age- and AD-related neurodegenerative processes and thus to clinical diagnosis. Indeed, prior work demonstrates that N-terminal containing fragments of tau appear to be actively secreted by neurons in response to Aβ exposure, whereas fragments without an intact N-terminus may be passively released by injured neurons^21,22^. Regarding clinical diagnosis, CSF and plasma measures of full-length and mid-region only fragments of tau do not appear to efficiently differentiate MCI and AD populations from unimpaired controls, whereas levels of N-terminal containing tau fragments can separate these populations^20^. Ongoing work elucidating the biology underlying this diversity of tau fragments should indicate how quantifying distinct tau fragments might be used to capture different aspects of the AD process and how analytes such as NT1 can be combined with phospho-tau measures to improve stratification and mark disease progression in clinical trials.

Though the PACC and similar measures are in use in observational studies and ongoing prevention trials, it is important to note that the PACC is weighted toward memory measures and may not fully capture early disruptions in other cognitive domains^25,26,42,43^. Given the potential for clinically variable symptomatic presentations of AD pathology (including language and visuospatial predominant forms), further work using additional, diverse cognitive tests is needed to determine the extent to which plasma NT1 may predict changes in executive, language, and visuospatial function on the path to Alzheimer’s disease.

While we observed that plasma NT1 was a statistically stronger predictor of cognitive decline and neurodegeneration than NfL in the present study, this result must be interpreted in the context of the study population and the use of plasma rather than CSF. HABS enrolls older adults who are clinically normal, many of whom are not on an AD (or other) neurodegenerative trajectory. As a result, there are relatively few participants who progress from normal to clinically impaired, an important limitation. It may well be the case that markers of neurodegeneration such as NfL may be stronger predictors of decline in already cognitively impaired populations^15,17,19,44–49^ (or in populations at very high genetic risk of decline^34^), and further work is needed to understand which blood-based biomarkers of AD pathobiology are most informative at different stages of disease.

Similarly, the lack of a significant association between plasma NT1 and cross-sectional (vs. longitudinal) FTP PET signal in the temporal lobe composite suggests that NT1 levels may reflect both tau- and non-tau mediated neurodegenerative processes. Alternatively, levels of NT1 may be capturing aspects of pathological tau that are not fully captured by tau PET (or that may be captured by tau PET signal at some later time). Further studies with greater length of PET follow-up and replication in other samples are needed to distinguish these possibilities and assess the extent to which measurement of this tau fragment is specific for AD- versus non-AD pathways to cognitive impairment.

However, the predictive data we obtained with NT1 as a plasma biomarker present clear advantages over CSF and brain imaging for serial measurement over many years using a non-invasive, inexpensive and widely applicable immunoassay. The drive toward earlier and earlier intervention in AD clinical trials has brought with it the critical need to enrich trials with individuals who are simultaneously minimally symptomatic and at high risk of cognitive decline over the course of a relatively short clinical trial. Accessible and inexpensive blood biomarkers such as NT1 that predict impending cognitive decline in the preclinical stages of disease and are practical for use in large screening samples are well-suited to achieve this critical enrichment and thereby accelerate the development of disease-modifying therapies for AD.

## Methods

### Participants

Cross-sectional and longitudinal data for this study came from participants in the Harvard Aging Brain Study (HABS). HABS is a longitudinal observational study of cognitive aging and preclinical Alzheimer’s disease based at Massachusetts General (MGH) and Brigham and Women’s (BWH) Hospitals in Boston, MA. All participants in this community-based convenience sample provided written informed consent prior to undergoing any study procedures. The HABS protocol is approved annually by the Partners Human Research Committee.

HABS participants undergo a comprehensive medical and neurological evaluation to ensure they have no exclusionary medical, psychiatric, or neurological conditions, including a recent history of alcoholism or drug abuse. Individuals with Modified Hachinski Ischemic Scores >4 and those with a history of stroke with residual deficits were excluded. At the time of study entry, all participants were judged to be clinically unimpaired, with clinical dementia rating (CDR) = 0, Geriatric Depression Scale score of <11, Mini-Mental State Status Examination score of 27 or greater, and performing within education-adjusted norms on the Logical Memory Delayed Recall. Baseline visits for the HABS studies take place over a 3- to 4-month period. Participants were required to have NT1, NfL, at least one full year of cognitive follow-up, baseline PiB PET, baseline structural MRI, APOE ε4 status, and demographic data to be included in the Main Dataset (Table 1). This resulted in a study population where 167, 45, 16, 5, and 3 participants had completed 6, 5, 4, 3, and 2 annual visits, respectively. Of the 236 participants included in the Main Dataset, 23 progressed to Mild Cognitive Impairment (20 of 23) or AD dementia (3 of 23) during the follow-up period. The diagnosis of MCI or AD was made by experienced physicians and neuropsychologists (blinded to biomarker data) as part of a clinical consensus conference. As 18F-Flortaucipir (FTP) tau PET was introduced mid-study, most HABS participants received FTP PET after their baseline visits. Thus, to assess relationships between 18F-FTP (tau) PET and NT1, a second dataset was generated (Tau dataset, Table 1). To be included in the tau dataset, participants were required to have plasma NT1, PiB PET, and FTP PET data within 1 year of each other and also have at least one additional FTP PET scan that took place after the initial FTP PET and NT1 measurement. This resulted in a dataset in which 79, 30, and 3 participants had 2, 3, and 4 FTP PET scans available for longitudinal analysis, respectively.

### Behavioral methods

HABS participants undergo cognitive testing annually, including cognitive measures sufficient to calculate the Preclinical Alzheimer’s Cognitive Composite (PACC5). The PACC5 is calculated by standardizing and combining scores from the Mini-State Mental Status Exam (MMSE)^50^, Weschler Digit Symbol Coding^51^, WMS-R Logical Memory Delayed Recall^52^, free + total recall from the Buschke Free and Cued Selective Reminding Test (fcSRT)^53^, and performance on the three category semantic fluency task (CAT)^25^. Higher PACC5 scores reflect better performance. PACC5 was used as the primary cognitive performance measure in the present study. Slopes and raw PACC scores were standardized prior to entry in models. Individual test measures were subsequently tested in exploratory, post hoc analyses to determine whether higher baseline plasma NT1 was associated with greater declines in particular cognitive domains (Fig. 1b–f).

### PET imaging methods

Fibrillar β-amyloid burden was assessed using 11C-Pittsburgh compound B (PiB) PET. Tau burden was measured using 18F-flortaucipir (FTP; previously known as AV-1451 or T807). PET imaging was carried out at the MGH PET facility (ECAT EXACT HR+ scanner; Siemens, Erlangen, Germany). Baseline PiB PET data used here was acquired during the first year of participation in HABS. Tau PET was introduced into HABS after the start of the study, with the majority of participants undergoing tau PET at year 4 of the study (Supplemental Table 1). Detailed HABS protocols for PiB and FTP PET have been previously published^28,29,54^. As in prior studies from our group, PiB PET measurements were represented as a distribution volume ratio across a composite of frontal, lateral temporal and parietal, and retrosplenial regions^27,29,54–57^. A temporal composite of FTP PET was calculated to reduce multiple comparisons, using the voxel-weighted average (temporal composite) FTP binding within five bilateral, FreeSurfer-defined, contiguous temporal lobe regions of interest (entorhinal, parahippocampal, inferior temporal, fusiform, and middle temporal cortices). Prior work indicates that these regions are early sites of tau accumulation^29^ and regions where tau PET signal is most highly correlated with PiB PET in HABS^58^. Cerebellar gray matter (as defined by FreeSurfer) served as the reference region for PiB and FTP PET. PiB and FTP PET data were corrected for partial volume effects using the geometric transfer matrix method^59^. Participants were dichotomized into high and low PiB PET groups based on baseline PiB PET measurements using a previously described, Gaussian mixture modeling derived cutoff value for the HABS sample^6,55^. To examine interactions between baseline plasma NT1 and PiB PET in participants with below cutoff values of PiB-PET signal (Low PiB group), a median split (above and below a PiB PET FLR SUVR of 1.18) was performed (Fig. 1b). Additional analyses used the Johnson–Neyman method (as implemented in the R package “interactions”) to identify a PiB-PET threshold at which the interaction of NT1 and PiB PET led to a statistically significant prediction of cognitive decline (Supplemental Fig. 1).

### MRI methods

MRIs were completed at the MGH Athinoula A. Martinos Center for Biomedical Imaging using a Siemens TIM Trio 3T System with a 12-channel head coil. T1-weighted multi-echo magnetization prepared rapid acquisition gradient-echo (MPRAGE) structural images were collected using the following scan parameters: repetition time (TR) = 2200 ms, echo times (TE) = 1.54, 3.36, 5.18, and 7 ms, flip angle = 7°, 4× acceleration, 1.0 × 1.0 × 1.2 mm voxels. MRI structural data in HABS are acquired at Years 1, 4, and 6. At the time of analysis, 195 of 237 participants included in the Main Dataset had more than one MRI available for longitudinal analyses (104 with 3 or more and 91 with 2; mean 2.53 ± 0.5 SD scans per participant). Estimation of cortical thickness and subcortical volumetric segmentation was performed with FreeSurfer version 6.0 (http://surfer.nmr.mgh.harvard.edu/). Longitudinal structural measurements were derived using the FreeSurfer longitudinal processing stream, a temporally unbiased segmentation approach that decreases noise in longitudinal analyses^60^.

### Plasma samples

All biochemical analyses were performed by investigators blinded to all demographic, clinical, and neuroimaging data. Baseline plasma samples were drawn during the first year of participation in HABS. All baseline plasma samples were collected following an overnight fast into EDTA containing tubes. Aliquots of plasma were stored at −80 °C prior to analysis. Only fasting baseline samples were used in the main dataset (Table 1). In the Tau dataset, NT1 values from the plasma sample closest in time to FTP PET imaging were used. We investigated whether fasting impacted NT1 values and observed no effect of fasting vs. non-fasting state on NT1 values (*t*(107) = 0.424, *p* = 0.673).

### Tau NT1 Simoa assay

Consumables and reagents other than antibodies were obtained from Quanterix (Lexington, MA). The tau capture antibody BT2 (194–198) was conjugated onto paramagnetic beads at 2 mg/mL. Detector antibody Tau12 was biotinylated according to the manufacturer using a ratio of 40 parts biotinylation reagent to 1-part antibody. Plasmas were centrifuged at 14,000 × *g* for 4 min and then diluted 1:4 with Tau 2.0 sample diluent reagent (Quanterix). Tau441 standard was diluted linearly with Tau 2.0 sample diluent to a concentration range of 540–0.02 pg/mL. Sample, standards and blanks were prepared in 1.5 mL low-binding Eppendorf tubes and were analyzed in triplicate.

The NT1 Simoa assay utilized a 3-step protocol and was performed at ambient temperature on a Simoa HD-1 analyzer (Quanterix Corp.). In step 1, 100 μl of standard, blank, or sample were added to beads coated with capture antibody and mixed for 30 min. The beads were then harvested and washed with wash buffer. In step 2, biotinylated detection antibody (0.6 μg/ml) was added and incubated for 10 min 30 s, and the beads were then washed three times. In step 3, 150 pM streptavidin-β-galactosidase was added, and following a further wash step, enzyme substrate (resorufin β-D-galactopyranoside) was added. The bead-bearing complexes were then resuspended and loaded into Simoa arrays, each containing 216,000 femtoliter-sized wells. The average enzyme unit per bead (AEB) was determined as described previously (3). Standard curves of AEB vs. Tau441 concentration were fitted to a five-parameter logistic function with 1/Y2 weighting.

### Preparation of recombinant tau

Escherichia coli strain BL21(DE3) was transformed with expression vectors pNG2/htau40, pNG2/N-terminal tau, and pNG2/C-terminal tau, respectively, encoding human tau441, N-terminal residues 2–230 lacking inserts 1 and 2 (i.e., residues 44–103), and a CT construct encompassing residues 231–441. Proteins were expressed and purified as previously described^20^. The purity of proteins was assessed by SDS-PAGE/sliver staining and reverse phase-high performance liquid chromatography. Concentrations were determined by absorbance at 280 nm and using predicted extinction coefficients for each tau construct (ε280 = 7575 and 4470 M^−1^ cm^−1^ for human tau441 and NT1, respectively).

### Quality control of tau NT1 assay

LLoQ is defined as the lowest back-interpolated standard with a signal recovery of 100 ± 20%, a signal 2× greater than background, and a coefficient of variance (CV) of ≤20%. Duplicate baseline plasma samples from the same subject were assayed in adjacent wells in the same run on one day to avoid inter-day variation. Internal control pooled plasma 7 (P7) was put in the first and the last assay tube in each day of assay. The P7 intra-assay variance ranged from 0 to 11%, and the inter-assay variance was 9%. Samples with CV < 10% on the initial assay were re-run the next day. The correlation between the 1st run and the repeat run for these samples was *R*^2^ = 0.92.

### Neurofilament (NfL) Simoa assay

Plasma was centrifuged at 14,000 × *g* for 4 min and diluted 1:4 with NfL sample diluent reagent (Quanterix), then assayed in duplicate on SIMOA kits (based on Uman Diagnostics UD1/mAb 47:3 and UD3/mAb 2:1 antibodies). Assays were performed according to the manufacturer’s instructions^61^, and manufacturer provided materials (including antibody bearing beads at the supplied concentration) were used. All sample CVs of duplicate measurements were <10%.

### Quality control of NfL assay

The lowest back-interpolated standard with a signal recovery of 100 ± 20%, a signal 2× greater than background, and a CV of ≤20%. Pre-diluted NfL standards run on 9 separate days produced stable LLoQs of 0.458 pg/mL. All samples from the same HABS subject were assayed in adjacent wells in the same run on a day to avoid inter-day variation. Internal controls 1 (CN1): pooled plasma #5 (P5) and pooled plasma #7 (P7) were analyzed on different locations in each assay/day. Inter-assay variance of control 1 = 6%. Intra-assay variance of P5 was 0–10%, and the inter-assay variance = 11%. Intra-assay variance of P7 was 0–9%, and the inter-assay variance = 11%. As with NT1, samples with CV < 10% across duplicates were re-run. All samples from the same participant were repeated together in adjacent wells; correlation of 1st and repeat runs was high (*R*^2^ = 0.83).

### Statistics

Linear and linear mixed-effects models were used to assess NT1 relationships to cognitive and imaging measures. These analyses were implemented using R version 3.4.4 (nlme package; R Foundation for Statistical Computing). As noted in the text, main analyses included age, sex, and APOE ε4 carrier status as covariates. Longitudinal analyses included these covariates both alone and including interactions with time, in addition to terms for random slope and intercept. Intracranial volume (ICV) was accounted for in structural MRI analyses, with ICV included as a covariate in GMV analyses and with ICV regressed out of HV prior to study entry. Models with interaction terms included all lower-order terms. Analyses in which cognitive variables were modeled also included years of education as a covariate. Two-tailed tests were used throughout. Unless otherwise noted, *p*-values shown are false discovery rate adjusted (FDR) to constrain type I error. 95% confidence intervals are shown in shaded gray in all figures.

Several sensitivity analyses were performed to assess possible confounds and aid in the selection of covariates. Seven participants in the Main Dataset (2.96% of 236 subjects) had been included as low Aβ controls in a prior report from our group that also examined plasma NT1^20^. To assess whether this very small degree of overlap impacted the findings here, we performed supplemental analyses in which these seven overlapping participants were removed from the dataset. We observed very similar associations between plasma NT1 levels and PACC decline, HV and GMV loss whether these seven participants were included or excluded from the analysis (Supplemental Table 3). A second set of sensitivity analyses assessed whether the presence of vascular risk factors impacted the association of NT1 with cognitive trajectories. Similar to prior reports from HABS, we used the office-based Framingham Cardiovascular Disease risk score (FHS-CVD)^62^ as a summary measure of systemic vascular risk. Consistent with prior reports from the HABS sample, higher FHS-CVD was associated with longitudinal PACC decline (*t*(1053) = −3.18, nominal *p* = 0.0015), but we observed that inclusion of FHS-CVD as a covariate did not weaken the association of baseline plasma NT1 levels with longitudinal PACC performance (Supplemental Table 4). Parallel sensitivity analyses demonstrated that neither self-reported race (grouped into White/Black/Other Non-white) nor socioeconomic status (operationalized using the Hollingshead Index^63^) altered the relationship of NT1 to longitudinal PACC decline (Supplemental Table 4).

### Reporting summary

Further information on research design is available in the Nature Research Reporting Summary linked to this article.

## Supplementary information

Supplementary Information

Reporting Summary

## Data Availability

Data from the Harvard Aging Brain Study is available online at http://nmr.mgh.harvard.edu/lab/harvardagingbrain/data. Longitudinal data will be made available in periodic data releases at the same web address or via direct request to the corresponding author(s). Requests for material, data, and correspondence can be addressed to Dr. Selkoe and/or Dr. Sperling. Qualified investigators must abide by the Harvard Aging Brain Study online data use agreement, designed to protect the privacy of our participants. Source data are provided with this paper.

## Source data

Source data

## Code availability

Imaging analysis was performed using FreeSurfer v6.0, available at https://surfer.nmr.mgh.harvard.edu/fswiki/DownloadAndlnstall and tools available at https://mrtools.mgh.harvard.edu/index.php?title=Main_Page. As described in the paper, a commonly used, open-source software (R v 4.0.0; Foundation for statistical computing) was used for statistical analyses.
