# Supplementary material for: Plasma N-terminal tau fragment levels predict future cognitive decline and neurodegeneration in healthy elderly individuals
Source: Nat Commun. 2020 Nov 27;11:6024. doi: 10.1038/s41467-020-19543-w (PMC7695712; doi:10.1038/s41467-020-19543-w)
Supplement: Supplementary file 1 — Supplementary Information [file 41467_2020_19543_MOESM1_ESM.pdf]

Supplementary Table 1: Baseline Predictors of Cognitive Trajectories (PACC Slope)			
	Cohen's d	95% CI	p-value
PiB PET	-0.91	(-1.19 to -0.64)	1.90×10^{-9}
Plasma NT1	-0.71	(-0.98 to -0.44)	4.44×10^{-6}
HV	0.61	(0.34 to 0.87)	4.45×10^{-5}

Corrected for age, sex, APOE ε4 status, and years of education. HV adjusted for intracranial volume. Two-tailed t-tests were used throughout. FDR-corrected p-values are shown.

Supplementary Table 2: Baseline plasma NT1 predicting decline (PACC slope), controlling for PiB PET and HV

	Cohen's d	95% CI	p-value
NT1 controlling for PiB PET	-0.64	(-0.91 to -0.37)	2.58×10^{-5}
NT1 controlling for HV	-0.68	(-0.95 to -0.42)	7.38×10^{-6}
NT1 controlling for HV and PiB PET	-0.63	(-0.90, -0.36)	3.33×10^{-5}

Corrected for age, sex, APOE ε4 status, and years of education. HV adjusted for intracranial volume. Two-tailed t-tests were used throughout. FDR-corrected p-values are shown.

Supplementary Table 3: Summary of Sensitivity Analyses I			
Dataset	Baseline Plasma NT1 Effect on:		
	PACC	GMV	HV
Main Dataset	t(1070) = -5.05, p = 4.42 x 10-7	t(292) = -2.19, p = 0.029	t(292) = -2.73, p = 0.0068
Main Dataset excluding participants in Chen et al.	t(1038) = -5.01, p = 5.44 x 10-7	t(284) = -2.05, p = 0.0423	t(284) = -2.79, p = 0.0057

Model: Outcome ~ Plasma NT1 + Covariates; age, sex, ApoE 4 status, years of education, and interactions with time included in all models; Chen et al., Alzheimer's & Dementia 2019; nominal p-values shown

Supplementary Table 4: Summary of Sensitivity Analyses II	
Dataset/Covariates	Baseline Plasma NT1 Effect on PACC
Main Dataset	$t(1070) = -5.05$, $p = 4.42 \times 10^{-7}$
Main Dataset excluding participants in Chen et al.	$t(1038) = -5.01$, $p = 5.44 \times 10^{-7}$
Main Dataset covarying FHS-CVD	$t(1054) = -4.66$, $p = 3.16 \times 10^{-6}$
Main Dataset covarying race	$t(1059) = -4.67$, $p = 3.01 \times 10^{-6}$
Main Dataset covarying Hollingshead Score	$t(1069) = - 5.02$, $p = 5.17 \times 10^{-7}$

Model: PACC ~ Plasma NT1 + Covariates; age, sex, ApoE 4 status, years of education included in all models; Chen et al., Alzheimer's & Dementia 2019; Race variable derived from self-report (stratified into White, Black, Other/Non White). Two-tailed t-tests were used throughout. Nominal p-values are shown.

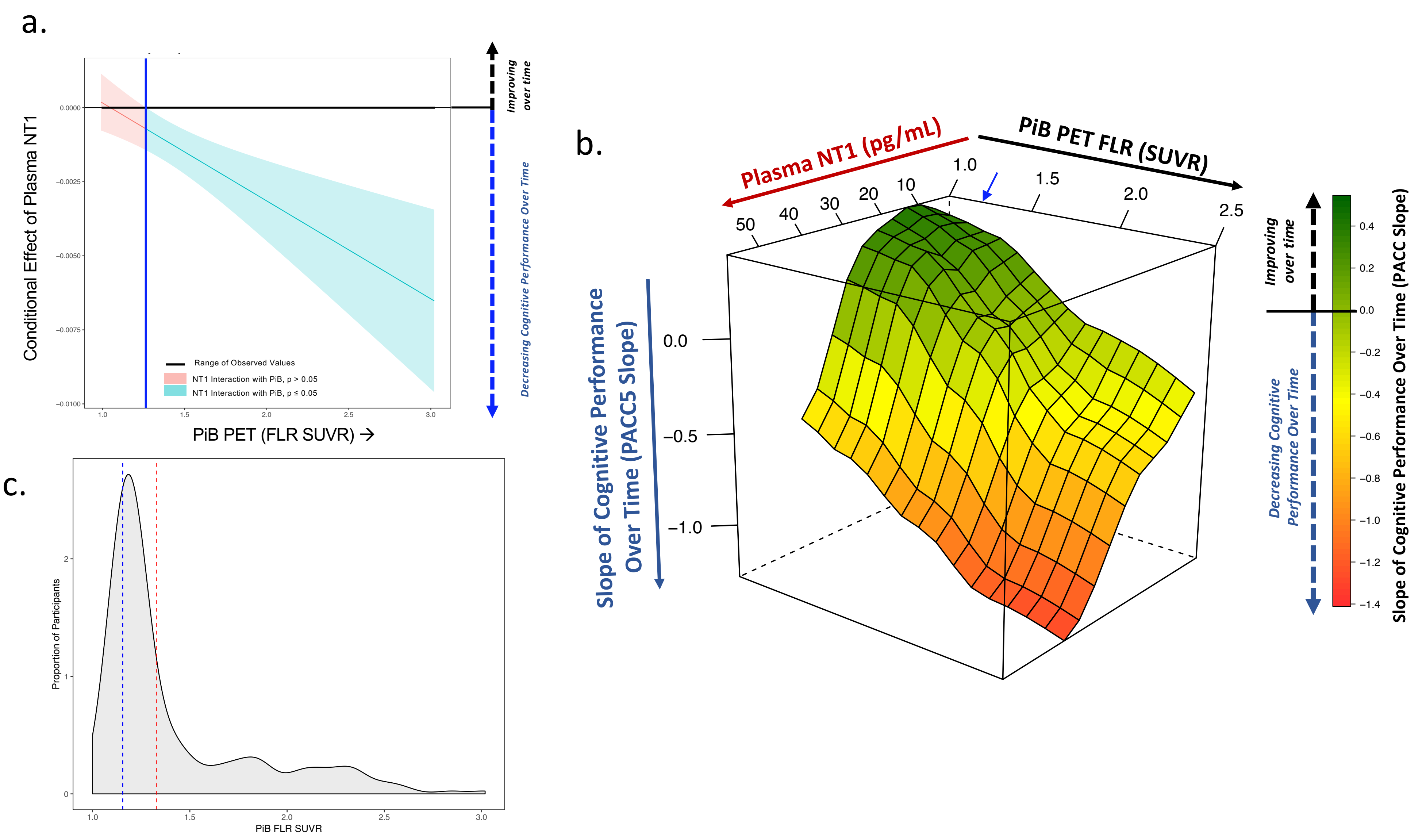

Supplemental Figure 1: *Baseline plasma NT1 interacts with even relatively low levels of baseline β-amyloid burden to predict cognitive decline.* Higher baseline plasma NT1 levels are predictive of decreased cognitive performance during longitudinal follow-up. A significant interaction is present between baseline NT1 and β-amyloid levels (measured by PiB PET), such that higher NT1 and higher PiB are synergistically associated with decreased cognitive performance over time. The PiB PET SUVR at which this interaction becomes statistically significant (at a nominal **α** of 0.05) corresponds to 1.16, as shown in the Johnson-Neyman plot in panel A and in the continuous interaction (”surface”) plot shown in panel B. The distribution of PiB FLR SUVR values in the study sample is shown in panel B. The interaction of NT1 and PiB with respect to cognitive decline is observable at PiB PET FLR SUVR of 1.16 (A: blue line; B: blue arrow; C: blue line) and above, a value that is well below the conventional threshold for high β-amyloid levels in this sample (PiB FLR SUVR ≥ 1.32; C: red line). Two-tailed t-tests were used in Panel A. Shaded regions in A represent 95% confidence intervals for the fit of the linear regression model.

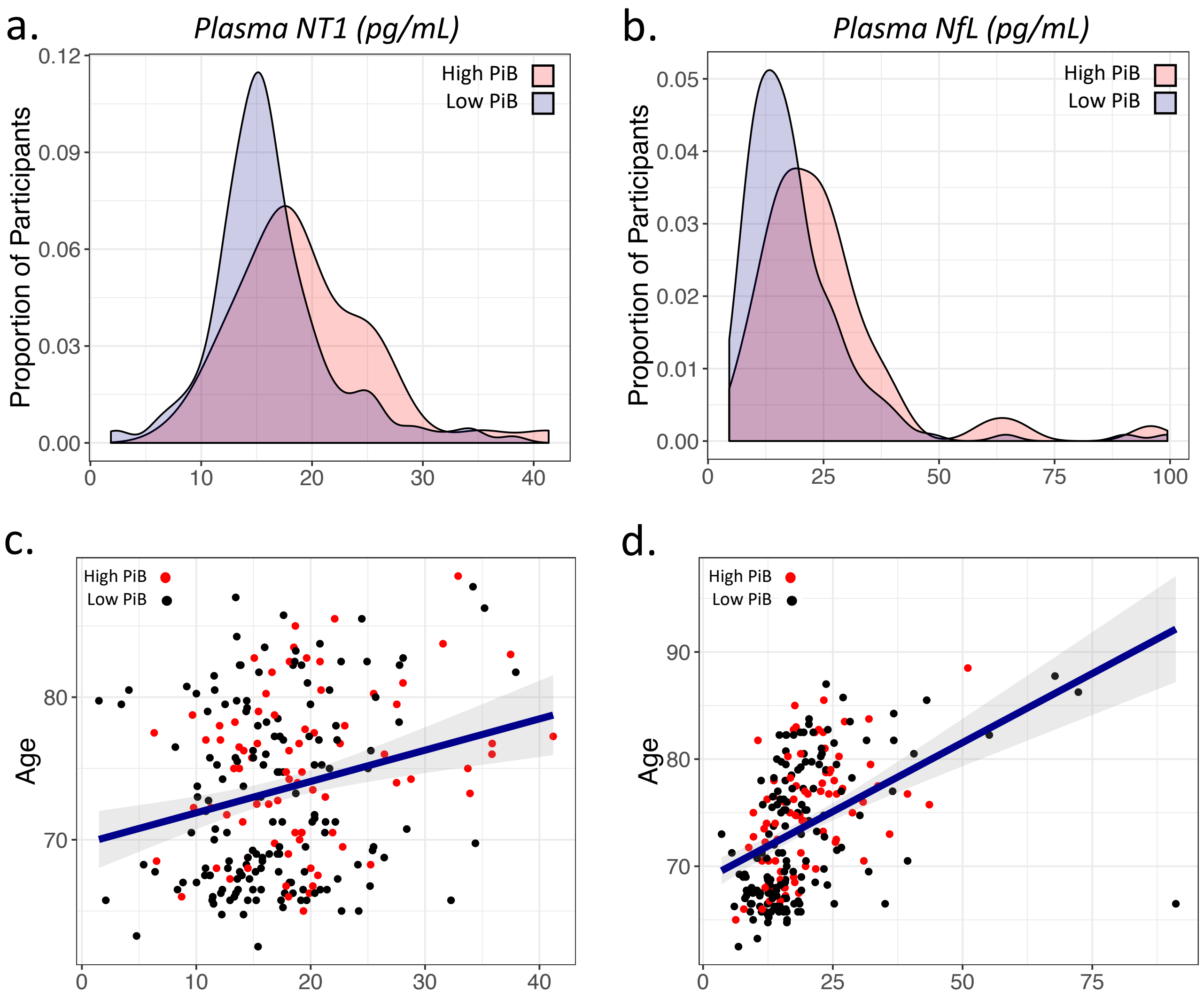

Supplemental Figure 2: *Distribution of plasma NT1 and NfL values by age and PiB PET.* Density plots of baseline plasma NT1 (A) and NfL (B) values separated by high and low baseline PiB PET. Both plasma NT1 and NfL were correlated with age, though this relationship was stronger for NfL ($r = 0.44$, $p = 1.33 \times 10^{-12}$) as compared to NT1 ($r = 0.24$, $p = 0.0002$). Plasma NT1 (C) and NfL (D) were both correlated with age, but this association was stronger for NfL. N = 236 for all panels. Two-tailed t-tests were used throughout. Nominal p-values are shown. Shaded regions in panels C and D represent 95% confidence intervals for the fit of each linear regression model.

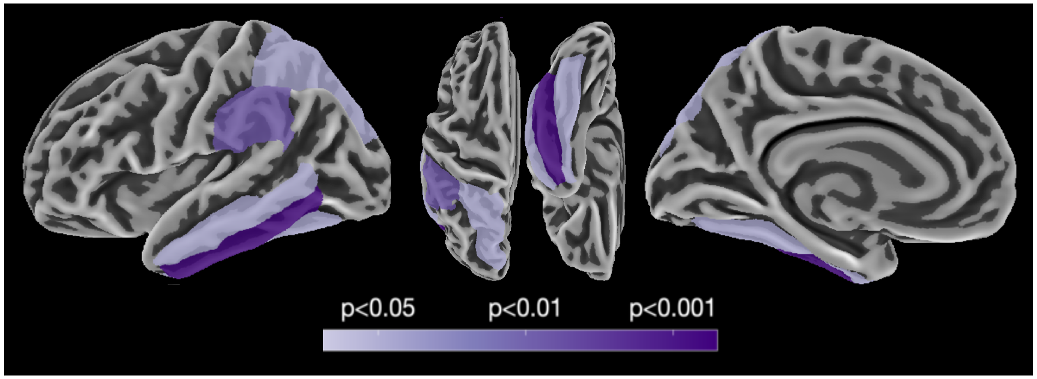

<u>Supplemental Figure 3:</u> *Regions of interest where NT1 and PiB PET are interactively associated with longitudinal changes in FTP (tau) PET signal.* An exploratory analysis depicting regions in which the interaction of plasma NT1 and PiB PET was significantly associated with longitudinal FTP PET signal. Greater NT1 and greater PiB PET were associated with greater increases in FTP PET signal in all regions shown. Two-tailed t-tests with nominal p-values are shown, coded by color (see scale).
