# Supplementary material for: Plasma N-terminal tau fragment levels predict future cognitive decline and neurodegeneration in healthy elderly individuals
Source: Nat Commun. 2020 Nov 27;11:6024. doi: 10.1038/s41467-020-19543-w (PMC7695712; doi:10.1038/s41467-020-19543-w)
Supplement: Supplementary file 2 — Reporting Summary [file 41467_2020_19543_MOESM2_ESM.pdf]

nature research

Corresponding author(s): Dennis Selkoe
Reisa Sperling

Last updated by author(s): Sep 13, 2020

Reporting Summary

Nature Research wishes to improve the reproducibility of the work that we publish. This form provides structure for consistency and transparency in reporting. For further information on Nature Research policies, see our Editorial Policies and the Editorial Policy Checklist.

Statistics

For all statistical analyses, confirm that the following items are present in the figure legend, table legend, main text, or Methods section.

n/a	Confirmed	
☐	☒	The exact sample size (*n*) for each experimental group/condition, given as a discrete number and unit of measurement
☐	☒	A statement on whether measurements were taken from distinct samples or whether the same sample was measured repeatedly
☐	☒	The statistical test(s) used AND whether they are one- or two-sided<br>*Only common tests should be described solely by name; describe more complex techniques in the Methods section.*
☐	☒	A description of all covariates tested
☐	☒	A description of any assumptions or corrections, such as tests of normality and adjustment for multiple comparisons
☐	☒	A full description of the statistical parameters including central tendency (e.g. means) or other basic estimates (e.g. regression coefficient) AND variation (e.g. standard deviation) or associated estimates of uncertainty (e.g. confidence intervals)
☐	☒	For null hypothesis testing, the test statistic (e.g. *F*, *t*, *r*) with confidence intervals, effect sizes, degrees of freedom and *P* value noted<br>*Give P values as exact values whenever suitable.*
☒	☐	For Bayesian analysis, information on the choice of priors and Markov chain Monte Carlo settings
☒	☐	For hierarchical and complex designs, identification of the appropriate level for tests and full reporting of outcomes
☐	☒	Estimates of effect sizes (e.g. Cohen's *d*, Pearson's *r*), indicating how they were calculated

Our web collection on statistics for biologists contains articles on many of the points above.

Software and code

Policy information about availability of computer code

Data collection	No software used for data collection
Data analysis	Imaging analysis was performed using FreeSurfer v6.0, available at https://surfer.nmr.mgh.harvard.edu/fswiki/DownloadAndInstall and https://mrtools.mgh.harvard.edu/index.php?title=Main_Page. As described in the manuscript, a commonly used, open source software (R v 4.0.0; Foundation for statistical computing) was used for statistical analyses.

For manuscripts utilizing custom algorithms or software that are central to the research but not yet described in published literature, software must be made available to editors and reviewers. We strongly encourage code deposition in a community repository (e.g. GitHub). See the Nature Research guidelines for submitting code & software for further information.

Data

Policy information about availability of data

All manuscripts must include a data availability statement. This statement should provide the following information, where applicable:

- -Accession codes, unique identifiers, or web links for publicly available datasets
- -A list of figures that have associated raw data
- -A description of any restrictions on data availability

IData from the Harvard Aging Brain Study is available online at http://nmr.mgh.harvard.edu/lab/harvardagingbrain/data. Longitudinal data will be made available in periodic data releases at the same web address or via direct request to the corresponding author(s). Requests for material, data, and correspondence can be addressed to Drs. Selkoe and/or Dr. Sperling. Qualified investigators must abide by the Harvard Aging Brain Study online data use agreement, designed to protect the privacy of our participants.

Field-specific reporting

Please select the one below that is the best fit for your research. If you are not sure, read the appropriate sections before making your selection.

[x] Life sciences [ ] Behavioural & social sciences [ ] Ecological, evolutionary & environmental sciences

For a reference copy of the document with all sections, see nature.com/documents/nr-reporting-summary-flat.pdf

Life sciences study design

All studies must disclose on these points even when the disclosure is negative.

Sample size

Data came from 236 participants in the main longitudinal data set and 112 in the Tau PET longitudinal data set. All data was from the Harvard Aging Brain Study, a longitudinal cohort study of preclinical AD and cognitive aging. No prior power analysis was performed.

Data exclusions

Participants were required to have plasma measures for NT1, NfL, at least one full year of cognitive follow-up, baseline PiB PET, baseline structural MRI, APOE e4 status, and demographic data to be included in the Main Dataset (Table 1). As 18F-Flortaucipir (FTP) tau PET was introduced mid-study, most HABS participants received FTP PET after their baseline visits. Thus, to assess relationships between 18F-FTP (tau) PET and NT1, a second dataset was generated (Tau dataset, Table 1). To be included in the tau dataset, participants were required to have plasma NT1, PiB PET, and FTP PET data within 1 year of each other and also have at least one additional FTP PET scan that took place after the initial FTP PET and NT1 measurement. All participants with requisite available data were included in the analysis.

Replication

Plasma assays were run in triplicate (NT1) or duplicate (NfL).

Randomization

None; there was no intervention.

Blinding

All cognitive, imaging, plasma, and genetic assays and analyses were performed by investigators and staff blinded to participant identity, AD biomarker status, and clinical data.

Reporting for specific materials, systems and methods

We require information from authors about some types of materials, experimental systems and methods used in many studies. Here, indicate whether each material, system or method listed is relevant to your study. If you are not sure if a list item applies to your research, read the appropriate section before selecting a response.

Materials & experimental systems

n/a	Involved in the study	
[ ]	[x]	Antibodies
[x]	[ ]	Eukaryotic cell lines
[x]	[ ]	Palaeontology and archaeology
[x]	[ ]	Animals and other organisms
[ ]	[x]	Human research participants
[ ]	[x]	Clinical data
[x]	[ ]	Dual use research of concern

Methods

n/a	Involved in the study	
[x]	[ ]	ChIP-seq
[x]	[ ]	Flow cytometry
[X]	[ ]	MRI-based neuroimaging

Antibodies

Antibodies used

All antibodies used are commercially available. NT1: Tau capture antibody BT2 (Pierce Thermo Scientific; Cat MN1010) was used together with detector antibody Tau12 (EMD Millipore; Clone 12,Cat MAB2241); NfL: UD1/mAb 47:3 (Uman Diagnostics; Cat 27016-100) and UD3/mAb 2:1 (Uman Diagnostics; Cat 27018-100) were used as part of the Quanterix Simoa HD-1 assay kit at manufacturer supplied concentrations (Cat 103186)

Validation

The specificity of Tau 12 (MAB2241 ;EMD Millipore) and BT2 have been examined in several prior reports from our group (Refs 1-3 below) and by the commercial providers of these commonly used antibodies (https://www.emdmillipore.com/US/en/product/Anti-Tau-Antibody-clone-Tau-12,MM_NF-MAB2241#documentation ; https://www.thermofisher.com/antibody/product/MN1010.html?CID=AFLCA-MN1010).

The NfL antibodies (UD1/mAb 47:3 and UD3/mAb 2:1 - both Uman Diagnostics) have been widely used. Two recent ELISA validation studies were published in 2019 (refs 4-5).

1. Mengel, D., Liu, W., Glynn, R.J., Selkoe, D.J., Strydom, A., Lai, F., Rosas, H.D., Torres, A., Patsiogiannis, V., Skotko, B., and Walsh, D.M. (2020) Dynamics of plasma biomarkers in Down syndrome: the relative levels of Abeta42 decrease with age, whereas NT1 tau and NfL increase. Alzheimers Res Ther; 12(1): pp. 27. PMCID: PMC PMC7081580
2. Chen, Z., Mengel, D., Keshavan, A., Rissman, R.A., Billinton, A., Perkinton, M., Percival-Alwyn, J., Schultz, A., Properzi, M., Johnson,

K., Selkoe, D.J., Sperling, R.A., Patel, P., Zetterberg, H., Galasko, D., Schott, J.M., and Walsh, D.M. (2019) Learnings about the complexity of extracellular tau aid development of a blood-based screen for Alzheimer's disease. Alzheimers Dement; 15(3): pp. 487-496. PMCID: PMC PMC6476313
3. Guix, F.X., Corbett, G.T., Cha, D.J., Mustapic, M., Liu, W., Mengel, D., Chen, Z., Aikawa, E., Young-Pearse, T., Kapogiannis, D., Selkoe, D.J., and Walsh, D.M. (2018) Detection of Aggregation-Competent Tau in Neuron-Derived Extracellular Vesicles. Int J Mol Sci; 19(3). PMCID: PMC PMC5877524
4. Hendricks, R., Baker, D., Brumm, J., Davancaze, T., Harp, C., Herman, A., Budingen, H.V., Townsend, M., and Fischer, S.K. (2019) Establishment of neurofilament light chain Simoa assay in cerebrospinal fluid and blood. Bioanalysis; 11(15): pp. 1405-1418.
5. Gaetani, L., Hoglund, K., Parnetti, L., Pujol-Calderon, F., Becker, B., Eusebi, P., Sarchielli, P., Calabresi, P., Di Filippo, M., Zetterberg, H., and Blennow, K. (2018) A new enzyme-linked immunosorbent assay for neurofilament light in cerebrospinal fluid: analytical validation and clinical evaluation. Alzheimers Res Ther; 10(1): pp. 8. PMCID: PMC PMC6389166

Human research participants

Policy information about studies involving human research participants

Population characteristics	The main sample consisted of 236 people (mean age 73.57+/-SD6.09y; 94M/142F; 169 ApoE e4- and 67 ApoE e4+) and the tau dataset consisted of 112 people (mean age 72.73+/-SD6.03y; 62M/50F; 80 ApoE e4- and 32 ApoE e4+)
Recruitment	HABS is a community based convenience sample in the Boston Massachusetts metropolitan area. We recruit cognitively unimpaired older adults for this study mainly through word of mouth and outreach events in the community. As the HABS protocols require multiple visits for imaging and cognitive and other testing, there is some subject burden associated with this study. Additionally, certain people do not wish to undergo PET or other imaging for personal or medical reasons. Over the course of the HABS study this far, we have noticed that our participants tend to have slightly higher than average educational attainment and there are slightly more women than men. These characteristics of the sample may impact the generalizablity of the results from HABS and similar studies.
Ethics oversight	The HABS protocol is approved annually by the Partners Human Research Committee (MassGeneral and Brigham).

Note that full information on the approval of the study protocol must also be provided in the manuscript.

Clinical data

Policy information about clinical studies

All manuscripts should comply with the ICMJEguidelines for publication of clinical research and a completedCONSORT checklist must be included with all submissions.

Clinical trial registration	NCT00900770
Study protocol	To facilitate data sharing and understanding of the data available in HABS, we recently published a synopsis of the study protocol for this observational study (Dagley et al., Neuroimage, Jan 2017; PMID 25843019). The full protocol is also available on request at https://habs.mgh.harvard.edu/researchers/request-data/ and further information is also available at https://habs.mgh.harvard.edu/researchers/data-details/
Data collection	Data collection for the HABS study started in 2009 and is ongoing. Recruitment is also ongoing. Data for the present study came from information collected between 2009 and 2019.
Outcomes	NA

Magnetic resonance imaging

Experimental design

Design type	Only structural MRI is included in the present report, no functional neuroimaging.
Design specifications	ADNI derived structural MRI sequences were acquired on a Siemens 3T Trio Tim MRI. Full details of our MRI protocol can be found at our data-sharing site: https://habs.mgh.harvard.edu/wp-content/uploads/2020/06/ADNI2_T1.pdf
Behavioral performance measures	NA, no fMRI used

Acquisition

Imaging type(s)	Structural MRI only
Field strength	3T
Sequence & imaging parameters	Repetition time (TR) = 2200 ms, echo times (TE) = 1.54, 3.36, 5.18, and 7 ms, flip angle =7°, 4x acceleration, 1.0 x 1.0 x 1.2 mm voxels. More details at https://habs.mgh.harvard.edu/wp-content/uploads/2020/06/ADNI2_T1.pdf and in (Dagley et al., Neuroimage, Jan 2017; PMID 25843019)
Area of acquisition	Whole brain

Diffusion MRI ☐ Used ☒ Not used

Preprocessing

Preprocessing software	NA; no pre-processing. Processing was performed through FreeSurfer 6.0 (https://surfer.nmr.mgh.harvard.edu/fswiki)
Normalization	NA
Normalization template	MNI
Noise and artifact removal	NA
Volume censoring	NA

Statistical modeling & inference

Model type and settings	The two primary structural measures used are total gray matter volume (Fig 3A and 5C) and hippocampal volume (Fig 3B). Analyses for these measures were linear models with the structural measure as the outcome, plasma NT1 or NfL interacted with PiB FLR PVC as the main predictor of interest, Age, Sex, and ApoE e4 status as covariates. As noted in the text, Figure 3C depicts an exploratory ROI-based analysis in which the structural (outcome measure) is the cortical thickness of a cortical, FreeSurfer defined region of interest and the independent variables the same as in the above models.
Effect(s) tested	NA

Specify type of analysis: ☐ Whole brain ☐ ROI-based ☐ Both

Statistic type for inference (See Eklund et al. 2016)	NA
Correction	NA

Models & analysis

n/a	Involved in the study
☒	☐ Functional and/or effective connectivity
☒	☐ Graph analysis
☒	☐ Multivariate modeling or predictive analysis
